# Grief and grief support needs in Canada: A mixed methods protocol

**DOI:** 10.1177/26323524251334180

**Published:** 2025-05-09

**Authors:** Susan Cadell, David Kenneth Wright, Naheed Dosani, Jacques Cherblanc, Lauren Breen, Samar Aoun, Lydia Sequeira, Katherine Kortes-Miller, Amit Arya, Kelly Anthony, Christian Boudreau, Holly Prince, Marney Thompson, Mary Ellen Macdonald

**Affiliations:** 1 Renison University College, University of Waterloo, ON, Canada; 2 University of Ottawa, ON, Canada; 3 St Michael’s Hospital at Unity Health Toronto, ON, Canada; 4 Université du Québec à Chicoutimi, Saguenay, QC, Canada; 5 Curtin University, Perth, WA, Australia; 6 Perron Institute, Perth, WA, Australia; 7 University of Western Australia, Perth, WA, Australia; 8 Centre for Addiction and Mental Health, Toronto, ON, Canada; 9 Lakehead University, Thunder Bay, ON, Canada; 10 McMaster University, Hamilton, ON, Canada; 11 University of Waterloo, ON, Canada; 12 Victoria Hospice, BC, Canada; 13 Island Health, Victoria, BC, Canada; 14 Dalhousie University, Halifax, NS, Canada

**Keywords:** grief, public health model of bereavement support, grief literacy, mixed methods

## Abstract

**Background:**

In their lifetime, every person will experience the loss of someone they care about. In Canada, the COVID-19 pandemic, the ongoing opioid crisis, and the discovery of unmarked graves at residential schools have brought this into particular focus. Research and theory in the area of grief have evolved over the years. Grief literacy challenges us to better understand and support grief in all aspects of our society. The Public Health Model of Bereavement Support was theorized and tested in Australia. The supports people seek are explored and the model identifies low, medium, and high categories of risk of prolonged grief disorder.

**Objective:**

The purpose of this study is to advance public health understanding of grief and its support. The specific research objectives are to (1) test the Public Health Model of Bereavement Support in the Canadian context and (2) build a grounded theory of grief support.

**Design:**

This project uses a sequential mixed methods design.

**Methods:**

A Canada-wide survey in English and French will produce data that will be used to empirically test the Public Health Model of Bereavement Support. In the second phase, the grounded theory of grief support centers on voices that have not been widely heard in grief research. The mixed methods then fully elucidate grief and grief support in Canada.

**Results:**

This is the first study internationally to test this model in a (post)pandemic context, in a jurisdiction that legally permits medical assistance in dying, and in a context with an opioid crisis.

**Conclusion:**

The findings will allow us to better understand grief and the current realities of grieving, which has the potential to enhance the wellbeing of the millions of Canadians who are grieving.

## Plain language summary

Grief and grief support needs in Canada

Loss will happen to everyone at some time in their life. Grief is the natural reaction to loss. Grief has been particularly highlighted because of the COVID-19 pandemic. In Canada, there is an ongoing opioid crisis and there has been confirmation of unmarked graves of Indigenous children at residential schools. All this has caused much grief. And yet, we know little about how many people are grieving in Canada and what kind of supports they do or would benefit from. Grief literacy challenges us to better understand and support grief in all aspects of our society. The Public Health Model of Bereavement Support from Australia measures how many people are at risk of complications in their grief when someone they care about dies. We are testing this model in Canada by doing a survey and then interviews. In understanding grief and grief supports in the Canadian context, we can better help those who are grieving.

## Background

Over our lives, most of us will experience the death of someone about whom we care deeply. Grief is a multidimensional reaction to any important loss.^1–4^ Death-related grief, specifically, is the focus of this project. While a universal human experience, people experiencing grief often do so alone.^5^ Grief in Canada, as in many societies, can be heavily stigmatized, which can compound loneliness.^6^ The results of this experience include increased healthcare utilization^7^ and increased risk of death, including suicide.^8^ An individual’s responses to grief are simultaneously biological, psychological, cultural, and social. Grief supports need to be similarly multi-dimensional. Contemporary grief support models and research increasingly challenge the limits of the stage-based and individualized theories proposed in the 20th century. Recent public health research, such as the Public Health Model of Bereavement Support,^9^ has demonstrated the need for civic societies to enhance their “grief literacy;”^10^ that is, to become more knowledgeable about grief and therefore better equipped to support everyday citizens grieving everyday deaths.

**Figure 1.**
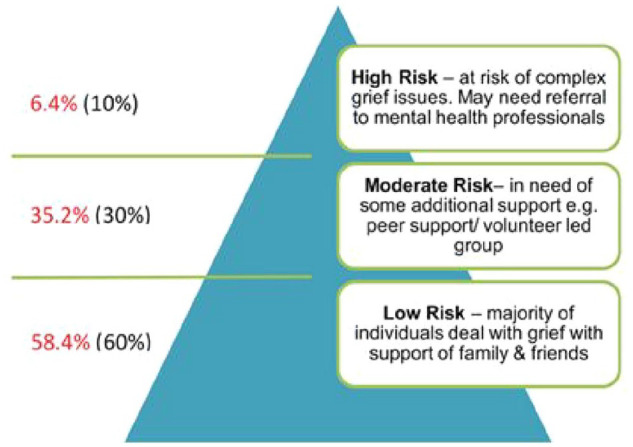
Public Health Model of Bereavement Support.^9^

The Public Health Model of Bereavement Support (see Figure 1)^9^ empirically establishes three tiers of bereavement support. The survey used in that study, which has been adapted for our project, collects details about the support that grievers seek and inquires about the helpfulness of those resources. This Australian scholarship demonstrated that all bereaved people should receive compassion and have access to information about bereavement and relevant local support. A smaller proportion, approximately 30%, should be offered more formal opportunities for reflection to normalize grief, advance the integration of the loss into the life story of the bereaved, and foster hope, meaning, and posttraumatic growth. Only a small proportion (approximately 10%) would benefit from specialist grief therapies aimed at targeting prolonged grief disorder (PGD) (p. 275).^9,11^ PGD involves severe and persistent grief that substantially interferes with functioning and quality of life.^12^ Cherblanc et al. reported 15% PGD during the pandemic in a French-speaking sample in Canada.^13^ Notably, the remainder – that is, up to 85% – of grievers who do not meet diagnostic criteria for PGD are largely ignored in the scholarly literature.

This public health approach to grief is novel and extremely important for three reasons. First, in this approach, grief is not conceptualized as a disease^14^; it is part of the human experience. Second, in our grief-denying society,^6^ grief is often positioned as problematic.^15,16^ While it is painful, not all grief is problematic or requires professional support. Offering professional support to some grievers *can actually do harm*. Aoun^17^ noted that it “may disrupt the natural course of grieving, . . .could interfere with support networks . . . or bereaved people may be prevented from finding their own solutions (p. 108).” Third, all grievers can be supported by family, friends, and social networks. While that may not be sufficient, professional forms of grief support should be seen as *additional.* Even for those receiving therapy for PGD, informal community support is still greatly needed.^17^

Thus, the Public Health Model of Bereavement Support has inspired an important rethinking of conventional grief support models in that it emphasizes linking the relevant level of intervention to grief support needs. Importantly, it has been tested in only two locations – Australia and Ireland.^16^ In Canada, statistics on grief were only recently collected in 2023.^18^ We know little about who could benefit from or need which services. From an equity lens, grief is likely experienced differently by various communities, particularly people who are experiencing structural vulnerability (e.g., poor, racialized).^19,20^

In addition to the potentially devastating personal and social consequences of grief, the landscape of death and grief is intensifying in Canada, making this research more urgent. Public health protection to curb the spread of COVID-19 has impacted how people die and grieve.^13^ The number of deaths due to opioids is skyrocketing,^21^ and there is rising inequality with the affordability crisis. Simultaneously, there are ongoing legislative shifts in Canadian end-of-life care, specifically related to medical assistance in dying (MAiD). The confluence of these events, within a broader social context in which grief is stigmatized and misunderstood, means that there has never been a more important time to understand and respond to grief, both at the individual and community levels.

## Purpose and objectives

The purpose of this study is to advance public health understanding of grief and its support. The specific research objectives are to (1) test the Public Health Model of Bereavement Support in the Canadian context and (2) build a grounded theory of grief support using public health grief theory^20^ as a sensitizing construct. We use mixed methods to achieve this purpose fully. By undertaking a Canada-wide survey in English and French, we are the first study to empirically test the Public Health Model of Bereavement Support in Canada and the first study internationally to test this model in a (post)pandemic context, in a jurisdiction that legally permits MAiD, and in a context with an opioid crisis. The grounded theory of grief support centers on voices that have not been widely heard in grief research. The mixed methods will then elucidate grief and grief support in Canada. Our innovative and interactive knowledge translation will distribute this knowledge through podcasts, webinars, social media, conferences, and publications. Overall, we will augment theoretical knowledge of grief to move from insights about individual, psychological experiences of grief to knowledge about how support can be better enacted at the interpersonal and community levels.

### Grief

Grief related to human death (i.e., bereavement) is the focus of this proposal. It is a typical response to death; it often begins before death occurs and can last for years.^22,23^ Bereavement and mourning are similar terms; we use grief to refer to the whole experience. In Canada, there are no accurate statistics about the number of grievers. Pre-pandemic, 260,000 people died each year.^24^ With an estimated average of four people bereaved for each,^25^ this means that, pre-pandemic, there were hundreds of thousands of grieving Canadians. The number of grievers has since risen in correlation with the number of additional deaths that have occurred because of the pandemic.^26^

Compounding a context of devastating loss, many forms of grief are stigmatized.^6^ Unlike in the case of spousal death (widow/er) or parental death (orphan), we have not evolved English words to describe people who are bereaved by the deaths of children, other family, or friends. This linguistic gap reflects a broader social attitude toward grief in which bereaved people in these circumstances are expected to grieve silently, out of the public eye.^27^

### Evolution of our understanding of grief

Over the last few decades, much has been written about grief and loss, dramatically shifting our understandings from simplistic, individualized notions to more socially embedded understandings. Kubler-Ross’s notion that grief involves five stages^28^ remains tenacious, recurring in popular culture as well as in health professions.^29–31^ Stage models have been thoroughly debunked,^32^ and theorists now work with more complex and sophisticated models, such as Stroebe and Schut’s Dual Process Model of Coping with Bereavement^33^ and Rubin’s Two-Track Model of Bereavement.^34^ Both of these models present a paradigm shift away from stage-based notions, normalize grief, acknowledge its longevity, and note its positive aspects. Overall, the field has moved away from the pathological view that sees “grief work” as needing to focus on severing ties with the person who died to recognize that growth and resilience are possible^35^ and that support should help establish a new kind of relationship: a continuing bond.^22,36^ In essence, scholars now articulate that grief is not a series of ‘stages’ to be worked through,^32^ a psychological disorder,^37^ or limited by any preset timelines around its resolution.^38^ By contrast, the death of someone important can fundamentally alter the very fabric of identity and personhood, which is not, in and of itself, a bad thing.^39^

### Grief support

Support for grievers can take many forms.^40–42^ It can come from family and friends being aware of those who are grieving, from neighbors dropping off casseroles, and from community associations organizing household chores. It can take the form of faith communities that know about the support needs of the grievers in their midst, and of professionals who are trained to understand nonpathological grief and deliver grief counseling and therapies.^1,29^ It can include national public education about grief,^43^ especially in schools and workplaces.^44–46^ One aim of this project is to better elaborate on what is helpful and what is missing along this entire spectrum.

### How grief is changing in Canada

Canadians have recently experienced changes in the ways people die and experience bereavement. One change is MAiD, which became legal in 2016 and has undergone legislative reform to expand access.^47^ MAiD is often framed through a lens of individual autonomy; however, its practice is deeply relational.^48^ Having to support a person’s request for MAiD can be burdensome for some family members,^49^ who often feel compelled to put their own needs and values aside to focus on helping their dying person achieve their wish. Knowing the date and time of death in advance, and counting down to that, carries an emotional intensity that grievers have described as “unprecedented” (p. 4)^50^ and deeply meaningful^50^ while remaining “heavy” and “enormous.”^51^ MAiD-related grief is also shaped by political polarization: Examples include grievers feeling unwelcome in palliative care spaces (which historically have opposed MAiD^52^) and keeping secrets about a MAiD death from their faith communities and social networks.^53–57^

Another change is the COVID-19 pandemic. Regardless of how people died (from the virus or other causes), public health measures greatly changed deathbeds and funeral gatherings.^54^ We have observed such consequences in our research. For example, in a study with nurses providing palliative care during the pandemic, Wright^52^ heard harrowing stories of people separated from their dying family members due to visitor restrictions. Furthermore, yet rarely mentioned, anyone who was already grieving before the pandemic experienced a reduction of in-person support for their grief. The numbers of pandemic grievers include those grieving deaths that occurred both inside and outside Canada. Such concerns fuel the Canadian Grief Alliance, convened by the Canadian Virtual Hospice, and this group’s ongoing lobbying for a national grief strategy.^57^

In addition, there has been a surge of deaths in Canada by unintentional poisonings, which include “accidental overdoses of various illicit drugs, prescription and over-the-counter medications, alcohol, as well as poisonings from solvents and pesticides (p. 1).”^58^ This crisis predates the pandemic; the increase in deaths disproportionately affects younger Canadians. These deaths are noted as having the “additional factor of being considered self-inflicted and may involve illicit activity. As such, they attract social stigma, which may extend to those left behind and marginalize and isolate them at a time when they most need support (p. 291).”^59^

Indigenous peoples experience tremendous grief due to ongoing colonization and structural violence.^60^ This has resulted in intergenerational trauma, struggles with addictions and mental health, poor holistic health outcomes, and the grief that is currently being augmented by the discovery of unmarked children’s graves at the sites of residential schools. Ongoing discoveries as well as other realities, such as overrepresentation in the child welfare system and the Missing and Murdered Indigenous Women and Girls Inquiry, exacerbate grief. Indigenous peoples are also disproportionately underserved by the health system and have little or no access to culturally safe grief support. COVID and societal inequities have magnified these grief experiences by eroding social systems and structures and magnifying racism and vulnerabilities within the health and social care system. For example, the grief experiences of family caregivers in Nunavik are compounded by multiple and ongoing losses, including those related to colonial policies and frequent deaths due to suicide and accidents. Current theoretical frameworks do not adequately address the prolonged and complex grief experienced by some Inuit, necessitating community capacity-building approaches and knowledge exchanges between Inuit and non-Inuit to develop culturally relevant and effective grief support strategies.^61^

### Prolonged grief disorder

Increasing attention is being given to prolonged grief as a diagnostic category.^62^ PGD is a term that describes severe and persistent grief that substantially interferes with functioning and quality of life.^12^ A meta-analysis found a pooled prevalence of 9.8% of grievers with PGD^63^; studies have reported a prevalence of up to 13.9%.^64^ The consequences of PGD include significant distress or disability, an inability to work, impaired social functioning, disrupted sleep, and increased suicidal tendencies.^65^ Importantly, the disproportionate attention in the scholarly literature to those experiencing PGD compared with the majority of grievers who do not reflect a narrow and pathological approach to grief.

### Grief literacy

Contemporary theoretical understandings of grief recognize that death, dying, and bereavement are normal parts of life; that the grief experience is unique to the individual griever; and that grief is a complex longitudinal experience that can continue for months or even years.^66^ While each experience of grief is unique and while different kinds of loss will beget different grief reactions, one element that seems to recur across grief experiences is loneliness and social isolation. *Grief literacy is* a social movement to create *grief-literate* societies.

Grief literacy directly augments the concept of death literacy. Death literacy emerged within *public health palliative care*, an academic and clinical rethinking of how palliative care should be expanded beyond clinical and institutional locations. Public health palliative care and the related “compassionate communities movement” seek to mobilize communities to take care of their citizens at the end of life.^17^ Grief literacy is the multidimensional capacity to access, process, and use knowledge about the experience of loss. The dimensions include the following: knowledge to facilitate understanding and reflection; skills to enable action; and values to inspire compassion and care. These dimensions connect and integrate individuals within sociocultural contexts. Situations that illustrate a grief-literate society are those in which people turn toward, rather than away from, one another in response to grief. The concept of grief literacy has implications for healthcare professional education. Cadell proposed that, in a grief-literate society, a lasting legacy of the pandemic should be “an increased ability to support one another in our grief.”^67^

### The Public Health Model of Bereavement Support

This model (Figure 1)^9^ was established by Aoun et al. in Australia to provide a “systematic and evidence-based framework for meeting the needs of bereaved family carers” (p. 14).^68^ For grievers for whom death had occurred 6 and 24 months prior, the theorized (black) and actual (red) proportions in each tier were very close.

Research shows that the practice of referring all grievers to therapy is ineffective and economically unsustainable and can do harm, including worsening the stigma of grief.^69,70^ While most people want human connection and support in grief, most grievers do not require specialized grief therapy.^71,72^ This model emphasizes “everyday assets” in the community that provide much of the care of the bereaved,^66,67^ and the importance of targeting support to need.

## Methods and analysis

### Overview of study design

This project uses a sequential mixed methods design.^73^ In sequential design, the quantitative and qualitative data of each strand are collected in sequence and analyzed separately. The different analyses are then combined to expand the breadth and depth of the overall results. After the data collection via the survey (described below), analyses will be run to identify the proportions of risk of PGD (low, medium, and high). Qualitative data generation commences with selection on the basis of those groups and other factors.

All procedures will be in accordance with the ethical guidelines of the participating institutions. All personnel working on the project will be graduate level and trained to deal sensitively with people who are grieving. Integrated knowledge translation, with a specific emphasis on verifying and sharing results with our community-based service provider collaborators, scholars, and the public, will occur throughout.

### Objective 1: Testing the Public Health Model of Bereavement Support

We will administer a population-based survey of bereavement support experiences in Canada, in English and French based on the questionnaire used in Australia.^9^ Closed- and open-ended questions ask about the support that the grievers sought and their helpfulness. This includes a measure of PGD to determine whether Canadians have similar results in terms of the risk of PGD. We will recruit 1000 respondents who are grieving any kind of death, 6–60 months earlier, to complete an online survey. A sample of 1000 will have over 95% power, assuming an effect size of 0.15, which is the one obtained by the Australian study. Six months following death is the earliest time period that a diagnosis of PGD can be established.^9,74^ We extended the timeframe to 60 months (instead of the 24 used in Stroebe et al.^8^) to acknowledge that the need for bereavement support often extends beyond 24 months.^38^ Extending the time frame allows us to be more inclusive of those who have experienced historical, intergenerational trauma, and grief^75^ that may not fit within the 24 months, as in Australia.^9^ We consider that significant grief experiences are not easily forgotten and that the need for support extends beyond 24 months.^38^

#### Survey tools

The Australian survey took 20–30 min to complete.^9^ We adapted it for the Canadian context, updated it with the PG-13-R,^76^ and translated it into French. Questions include demographics; supports desired, accessed, and not accessed; perceived needs; and whether these needs were met. The PG-13-R measures responses to separation; social/functional impairment; and cognitive, emotional, and behavioral symptoms. Five conditions must be met to indicate the presence of PGD: event (bereavement); separation distress; duration of distress (>6 months); cognitive, emotional, and behavioral symptoms; and social/occupational impairment. The score range is 11–55, and a score of 36 or more is a clinical indicator of PGD.^9^ The PG-13 exists in French; we have used that translation to create the revised version. We expect the time required to take the survey to be 20–30 min, as in Australia.

#### Recruitment

Recruitment began June 25, 2024, and is expected to close September 31, 2025. To obtain as many grievers within the timeframe as possible, participants are recruited via various methods. Dedicated websites (English https://uwaterloo.ca/grief-in-canada/ and French https://uwaterloo.ca/deuil-au-canada/) have been created. Instagram, LinkedIn, and Facebook accounts are being used, with both advertisements and boosted posts.^77^

Team members have considerable expertise in recruiting within 2SLGBTQ+ communities, with Indigenous people and people of color. Specific members are tasked with ensuring that equity, diversity, and inclusion considerations are integrated into all stages of the project. Team members and collaborators assist us using their usual approach with their contacts, be they service users, followers on social media, or communities of practice. These approaches could include verbal announcements, email blasts, or newsletters. All give priority to the recruitment of grievers who are Indigenous, racialized, LGBTQ2S+, gender diverse, and disabled through the use of inclusive language and images; this is because White, cisgender women’s experiences are overrepresented in grief research.^78^ The aim of using a variety of approaches is to reach as many potential participants as possible, from coast to coast to coast, not just those who have used our collaborators’ services or have access to social media throughout Canada.

Recruitment materials invite potential participants to complete an online survey. Alternatively, potential participants may request a paper copy of the survey or ask questions via a dedicated project email. The study process is explained on the website. We also ask if they would like to be considered for an interview. These approaches have been used successfully in prior research^35,79^ and are intended to minimize the added burden on individuals who may already be experiencing significant stress.

We have engaged the Survey Research Centre at the University of Waterloo to administer the survey. Data from paper copies will be manually entered into the database. Data collected by the Center are maintained on a secure, dedicated server, and protected by university firewalls. Files are backed up on a separate, secondary server located in a different area to ensure that data are never lost.

#### Analysis

We use the following analyses to answer the specific questions of Objective 1:

*Q1. How do the proportions of risk for PGD compare to the model?* Using the validated measure of PGD (PG-13-R),^76^ the 1000 respondents will be classified into three groups (low, moderate, and high risk) to test whether the proportions of Canadian grievers in each are the same as those in Figure 1. Assuming an effect size of 0.15, as was obtained by the Australian study, the corresponding chi-square test with a sample of 1000 will have over 95% power. We will then estimate the proportions of grievers in each of the three groups by computing 95% confidence intervals (CIs). With an expected proportion of the high risk of PGD at 10%, the sample of 1000 will have a precision of approximately ±2% (i.e., the width of the 95% CI will be approximately 4%). Assuming that the proportions of those at moderate and low risk will be 30% and 60%, respectively, the same sample will have a precision of ±3% for both categories.
*Q2. How do personal and support factors influence the risk of PGD?* This question involves the investigation of various statistical predictors of PGD. We will use generalized estimating equations (GEE) to model the effect of the covariates. Potential covariates are cause of death (e.g., illness, overdose, suicide), relationship to the deceased, age, gender, and helpfulness of services. We will also investigate interactions to obtain a better understanding of grief. We will first focus on a GEE model where the binary outcome variable is high versus medium/low risk. Standard model-building techniques (e.g., stepwise regression and Akaike information criterion) will be used to find the model with the best fit. Though binary GEE models will allow us to better understand the important high-risk group (vs medium/low risk) in a simpler, more easily interpretable model, we will also fit multinomial GEE regression models where the categorical outcome variable is high versus medium versus low risk. These various approaches will allow us to obtain a deeper, more nuanced understanding of PGD.

### Objective 2: Developing a grounded theory of grief support

The foundations of grief scholarship are rooted in an historical and systematic overrepresentation of the experiences of White middle-class women.^78^ This legacy continues; for example, when ‘parental’ experiences of child death are studied, it is typically mothers’ voices that predominate.^80^ Moreover, current conceptualizations often frame grief as an individual and psychiatric/medical phenomenon, eclipsing the many ways that grief is socioculturally determined.^78^ Given these limitations, it is important to advance theoretical understandings of grief that are empirically informed and that reflect (a) its diverse, intersubjective, and *relational* nature and (b) augment the *diversity of voices* that can inform these understandings. Relationality means exploring the relationships between the griever and their everyday social networks, their wider sociopolitical contexts, and with the person who has died. Diversity of voices means being intentional about inviting perspectives of grievers whose social location has been overlooked in much of the research to date (e.g., LGBTQ2+ people, people of color, Indigenous peoples) and who are grieving deaths that are less well researched (e.g., grieving overdose deaths, by MAiD, by colonial practices). By framing grief and grief support as diverse, relational phenomena, we can push our conceptualizations beyond narrow individualism and toward more socially informed understandings.

Given our intent to contribute to theory with this project, we selected grounded theory as the methodology to guide this objective. Grounded theory is explicit in its overall aim to produce theoretical knowledge through inductive analysis of qualitative data. Multiple versions of grounded theory have been articulated, reflecting differing epistemological perspectives about the creation and interpretation of research data and the role of the researcher. Our activities align with the *constructivist* grounded theory of Charmaz,^81^ which requires researchers to be explicit about their views and standpoints in relation to the research topic and to engage in a process of cocreation of research data with participants. An emphasis on positionality as well as reflexivity is important. Our team consists of diverse individuals in terms of Indigeneity, race, gender, sexual orientation, profession, and lived experience of grief. From these different positions, we are united in our commitment to compassionate understanding and enhanced support for *all* grievers within civic society.

We aim to create a *grounded theory of grief support* that explicates how people grieving different kinds of deaths, embodying diverse positionalities, feel (un)supported within their relationships of significance. Grief literacy, as a core concept of public health grief theory, serves as our “sensitizing concept,” which is “a starting point for data analysis and function[s] as an analytic lens or interpretive mechanism throughout the process (p. 2).”^82^ As soon as data generation begins, including analyzing the data generated from the open-ended questions in the survey, we will ask analytic questions about our data informed by this concept. For example, what do participants’ stories about their grief reveal about the knowledge, skills, and values of those who seek to support them? How do the dynamics of grief and grief support, as lived within participants’ relational contexts, reflect, and/or challenge normative ‘rules’ for appropriate grieving^10^?

The interview participants will be recruited from among the survey participants who agree to an interview. The selection of people will be purposive, prioritizing participants along the following axes: (1) grievers who demonstrate a low, medium, and high risk for PGD according to their survey responses; (2) people grieving deaths that have occurred during the pandemic, are the result of an opioid overdose or drug poisoning, or happened through MAiD; and (3) people who are not straight, cis, and White. We anticipate a total sample of 36–45 participants. This means that 12–15 individuals are recruited from each of the three risk categories for PGD; this may vary depending on the survey results. Demographic information from the survey will be used to ensure representational diversity (e.g., type of death, social positioning) within each set of participants.

The semi-structured, hour-long interviews will be conducted by one of the PIs or trained PhD trainees and take place online in French or English. The participants will be invited to bring a photo or other meaningful artifacts with them to the interview. This method can be useful for facilitating meaningful conversation and deeper understanding in qualitative research, including constructivist grounded theory.^83^ During interviews, we will ask participants to tell us about the person who died and what it has been like to grieve that person. We will probe for details around actions and processes that cause participants to feel (un)supported in their grief, focusing on relational dynamics at both the local and systemic levels.

French transcripts will be translated. The analysis will be led by the co-PIs. It will involve multiple readings and discussions about interview content and will be informed by the core principle of *constant comparison*.^84^ This means continuously scrutinizing aspects of our data in relation to other aspects (within and across interviews), surfacing areas of similarity, difference, and tension. Constant comparison will occur via a systematic approach to working with the data. We will move from more descriptive (line-by-line) to abstract (focused, axial) codes and engage in memo-writing (analytic reflections written, shared, and discussed within our team). As data are generated and analyzed, we will develop preliminary theoretical categories and hypothesize relationships between these categories. As analytic categories are developed, we will also continuously review the academic literature about grief and grief support to more fully account for areas of our emerging theory that are similar or different from what is already known and what is novel. This process of engaging with the literature during the analysis will also inform judgments about the stage of completion of our analysis. Decisions about ending our analysis will be made based on *saturation*. Although this concept is widely used – often inappropriately – as a generic marker of interpretive adequacy for diverse approaches to qualitative research, its origins are specific to grounded theory. Saturation here does not mean saturation of data; it means saturation of concepts.^85^ In other words, we will have achieved saturation when the analytic categories we create from our data and the relationships between them are well supported by ample and rich data that holds variation, and nuance.

### Meeting the overall aim to advance the public health understanding of grief and its support

We will use mixed methods analysis to expand our understanding of grief. The sequential mixed methods aim to better understand the experiences and support needs of grievers by integrating results from quantitative and qualitative data. Our methods are sequential in that the quantitative arm is first and informs the qualitative. Analyses will begin early with an examination of the first 100 respondents to the survey to determine who we may want to interview. Such sampling will continue throughout. We will make comparisons between our quantitative and qualitative data by *following the threads*^86^ that come through in our survey data and then exploring these threads for patterns, context, nuance, and discrepancies in our qualitative data. Analysis activities will enable interpretations that are faithful to the inherent complexities of the grief experience and that are richer than what can be captured through either method alone. For example, our quantitative data might suggest that those grieving a certain type of death appear to be at greater risk of PGD or patterns concerning gender and grief. Our qualitative data would then contain the narrative detail required to unpack and elaborate on this difference while allowing for the possibility of healing narratives.^87^

Where applicable, the guidelines found in Standard protocol items: Recommendations for interventional trials (SPIRIT) guidelines were followed for the protocol for this study^88^ (Supplemental Checklist). The final results of this mixed methods approach will be based on the integration of both datasets, which is an important benefit of this design.

## Knowledge translation

Integrated knowledge translation^89^ will occur throughout our project. We have strong relationships with each of our collaborators, with whom we have already begun to work to develop webinars and podcasts on the basis of what is already known about grief. Continuing these efforts is an effective way of accomplishing knowledge translation. We will also produce traditional academic outputs (e.g., journal articles and conference abstracts).

## Feasibility, challenges, and timeline

This research focuses on the experiences of those who are grieving a death anywhere in Canada. Some challenges of working with vulnerable populations in this area of research are the sensitivity required on behalf of the researchers to ethically navigate study activities and develop successful relationships with participants. All team members have this experience. The recruitment of 1000 bereaved people may pose a challenge. Team members have experience recruiting grievers.^6,22,71,90^ We have consistently found that grievers have a desire to share their experiences.

## Ethics

The survey in Objective 1 was approved by the University of Waterloo, Dalhousie University, and the University of Ottawa. Data collection began in this phase. Ethics approval is being sought from the same universities for Objective 2 (qualitative data generation).

## Discussion

This research will greatly contribute to public health understanding of grief. This approach is novel in several aspects. First, by taking a public health approach to grief, Aoun et al.^71^ noted that “for the field of bereavement policy and practice, a public health approach is in its infancy (p. 10).” Thus, this study will advance the field by providing empirical evidence to enhance policy and practice. Second, by testing the Public Health Model of Bereavement Support in Canada and its changing landscape, including with Indigenous peoples, the opioid crisis, MAiD, and COVID-19. Third, grounded theory will build a new theory of grief support, privileging voices that are not often heard. Fourth, the mixed methods will deepen our understanding of a public health approach to grief, helping our aim to elaborate this approach. Finally, innovative knowledge translation has the potential to inform the ability of bereavement services, community organizations, and informal networks to prioritize the care of specific grievers, according to each level of need.

## Conclusion

As our understanding of grief improves, we will be better able to design education and interventions in the future. These activities should range from the individual level to the national level and include all grievers, according to their needs, and assist in operationalizing the promise of developing compassionate communities in Canada. This research contributes to the field and enhances the quality of life of millions of Canadians who are grieving.

## Supplemental Material

sj-docx-1-pcr-10.1177_26323524251334180 – Supplemental material for Grief and grief support needs in Canada: A mixed methods protocol Supplemental material, sj-docx-1-pcr-10.1177_26323524251334180 for Grief and grief support needs in Canada: A mixed methods protocol by Susan Cadell, David Kenneth Wright, Naheed Dosani, Jacques Cherblanc, Lauren Breen, Samar Aoun, Lydia Sequeira, Katherine Kortes-Miller, Amit Arya, Kelly Anthony, Christian Boudreau, Holly Prince, Marney Thompson and Mary Ellen Macdonald in Palliative Care and Social Practice
